# Acupuncture for oligospermia and asthenozoospermia: A systematic review and meta-analysis

**DOI:** 10.1097/MD.0000000000027816

**Published:** 2021-12-03

**Authors:** Wen Jia, Chuan Wang, Ying Yin

**Affiliations:** Acupuncture Department, Wuhan Municipal Hospital of Integrated Chinese Medicine and Western Medicine, Wuhan, Hubei, China.

**Keywords:** acupuncture, asthenozoospermia, meta-analysis, oligospermia, randomized controlled trial, review

## Abstract

**Background:**

Acupuncture is widely used for oligospermia and asthenozoospermia in China, but its effect is unclear. We aimed to determine the effectiveness and safety of acupuncture in treating oligospermia and asthenozoospermia.

**Methods:**

An electronic search for randomized controlled trials evaluating acupuncture treatment in patients with oligospermia and asthenozoospermia published from database inception to October 2018 was conducted in PubMed, EMBASE, the Chinese Biomedical Literature Database, the Chinese Scientific Journal Database (VIP Database), the Wan-Fang Database, the China National Knowledge Infrastructure and the Cochrane Library. We established search terms related to 3 areas (oligospermia, asthenozoospermia, and acupuncture). Two authors independently screened all identified citations and extracted the data. The methodological quality of the included trials was assessed using the Cochrane criteria.

**Results:**

Seven studies with a total of 527 subjects were screened according to inclusion and exclusion standards, and most of the studies had significant methodological weaknesses. Seven randomized controlled trials tested the effects of acupuncture compared with placebo acupuncture and conventional medications in patients with oligospermia and asthenozoospermia. The results of this study suggest that acupuncture alone has no clear superiority in improving sperm motility (standard mean difference [SMD] = 1.13, 95% confidence interval [CI]: −0.64 to 2.89), the sperm concentration (SMD = 0.32, 95% CI: 0.27–0.92) or semen volume compared with placebo acupuncture. No significant difference was found between acupuncture alone and conventional medications in improving sperm motility (SMD = −0.53, 95% CI: −2.54 to 1.48), the sperm concentration (SMD = −1.10, 95% CI: −1.48 to −0.72) or semen volume. However, adjuvant acupuncture may enhance the effect of medications on improving sperm motility (SMD = 4.10, 95% CI: 1.09–7.12) and the sperm concentration (SMD = 1.07, 95% CI: 0.739–1.40), but the study heterogeneity was too high to establish robust conclusions.

**Conclusion:**

These results suggest that the current evidence does not support acupuncture as an effective treatment for oligospermia and asthenozoospermia; therefore, acupuncture is not currently recommended as a treatment for these conditions. However, owing to the high risk of bias among the included studies, the evidence is limited, and more large-scale, high-quality clinical trials are needed in the future.

**Trial registration number:**

PROSPERO CRD42018083885

## 1 Introduction

Because of harmful factors such as life pressures, worsening environments, and food contamination,^[1,2]^ the incidence of infertility has increased annually worldwide.^[3]^ Statistical results show that the infertility rate has reached 15% among people of childbearing age,^[3]^ with 50% of cases attributable to male infertility.^[4]^ Oligospermia and asthenozoospermia are common causes of male infertility. The World Health Organization defines oligospermia and asthenozoospermia as “a state of impaired sperm production and motility”.^[5]^ The main therapeutic strategy is drug treatment, including androgens, gonadotropins, corticosteroids, follicle-stimulating hormone, and antioxidants.^[6]^ Systematic reviews have shown that some treatments are effective, but others are not.^[7,8]^ China announced that the iconic one-child policy had finally been replaced by a universal two-child policy in 2015, resulting in a large birth demand among Chinese parents.^[9]^ However, the quality of sperm has been decreasing markedly in China.^[2]^ Meanwhile, a global decline in human sperm quality including low sperm production, inferior morphology, and poor motility has been noted in recent decades,^[10]^ and the causes vary and are complex.^[11]^ To increase the probability of pregnancy, many people turn to complementary and alternative medicine therapies.^[12]^

Acupuncture is an important component of complementary and alternative medicine,^[13]^ and some studies have suggested that acupuncture is a promising treatment for oligospermia and asthenozoospermia.^[14]^ A systematic review concluded that acupuncture might improve semen quality, but the evidence was insufficient because of the small number of studies.^[15]^ However, the review did not comprehensively search research papers in Chinese databases and used improper control groups (such as patients receiving Chinese herbal medicine and Chinese patent medicine), complicating objective evaluation of the clinical efficacy and safety of acupuncture in oligospermia and asthenozoospermia, and the previous review was also outdated; consequently, the effectiveness and safety of acupuncture for oligospermia and asthenozoospermia have not been verified. Despite limited high-quality evidence for the clinical efficacy of acupuncture in oligospermia and asthenozoospermia, acupuncture has been widely used in an attempt to reduce clinical symptoms and improve the quality of semen in China; therefore, the current research evidence must be summarized and analysed.

Therefore, the aim of this study was to assess the evidence from randomized controlled trials (RCTs) for acupuncture as a treatment method for patients with oligospermia and asthenozoospermia.

## 2 Methods

This is a systematic review, and ethical approval was not necessary. The protocol of this review was registered in the International Prospective Register of Systematic Reviews, and the trial registration number was CRD42018083885.

### 2.1 Literature search

We searched PubMed, EMBASE, the Chinese Biomedical Literature Database, the Chinese Scientific Journal Database (VIP Database), the Wan-Fang Database, the China National Knowledge Infrastructure and the Cochrane Library for relevant studies published prior to October 30, 2019. Studies published in Chinese or English languages were retrieved using a combination of keywords and subject terms. We established search terms related to 3 areas (oligospermia, asthenozoospermia, and acupuncture). The search strategy used in the PubMed database is shown in Table 1. We identified additional relevant articles by manually searching the references.

**Table 1:** Search strategy used in PubMed.

Number	Search items
1	Randomized controlled trial.pt
2	Randomized.ti,ab
3	Randomly.ti,ab
4	Groups.ti,ab
5	Trial.ti,ab
6	Or 1 to 5
7	Acupuncture.ti,ab
8	Electroacupuncture.ti,ab
9	Scalp acupuncture.ti,ab
10	Three edged needle.ti,ab
11	Fire needle.ti,ab
12	Auricular acupuncture.ti,ab
13	Dry needling.ti,ab
14	Warm acupuncture.ti,ab
15	Pyonex.ti,ab
16	Manual acupuncture.ti,ab
17	Or 7 to 16
18	Oligospermia.ti,ab
19	Low sperm count.ti,ab
20	Hypospermatogenesis.ti,ab
21	Hypospermatogeneses.ti,ab
22	Disc herniation-induced sciatica.ti,ab
23	Low sperm counts.ti,ab
24	Sperm count, low.ti,ab
25	Sperm counts, low.ti,ab
26	Oligoasthenoteratozoospermia.ti,ab
27	Oligoasthenoteratozoospermias.ti,ab
28	Oligozoospermia.ti,ab
29	Or 18 to 28
30	Asthenospermia.ti,ab
31	Astheno teratozoospermia.ti,ab
32	Astheno teratozoospermias.ti,ab
33	Teratozoospermia, astheno.ti,ab
34	Teratozoospermias, astheno.ti,ab
35	Asthenoteratozoospermia.ti,ab
36	Asthenoteratozoospermias.ti,ab
37	Or 30 to 36
38	6 and 17 and 29 and 37

### 2.2 Study selection

We included all RCTs that used acupuncture for oligospermia and asthenozoospermia. The patients conformed to the diagnostic criteria established in the management guidelines for male infertility issued by the authoritative diagnostic organizations. Any types of acupuncture were included, including manual acupuncture, scalp acupuncture, auricular acupuncture, electropuncture, three-edged needle acupuncture, warm acupuncture and dry needling. The control groups included patients receiving placebo acupuncture, no treatment controls, or patients receiving other conventional drugs, such as androgens, gonadotropins, corticosteroids, folliclestimulating hormone and antioxidants. Acupuncture with active medicine was also included if the medicine was also applied to the control group. The primary outcomes were the indicators of semen motility and sperm morphology). The secondary outcome was adverse events due to the treatments. We excluded the following studies: (1) non-RCTs; (2) duplicate studies; (3) studies lacking data integrity; (4) studies of acupuncture combined with Chinese medicine; and (5) studies without the information of interest.

### 2.3 Data extraction and management

Two investigators [Wen Jia and Chuan Wang] independently investigated the titles, abstracts and full texts of the papers identified through database searches to confirm that they contained eligible trials. The investigators extracted the data based on a standard data collection table. The main extracted information included the first author, publication year, country, age, gender, sample size, interventions, follow-up period, outcomes and adverse events. All disagreements were resolved by a third author [Ying Yin]. All retrieved studies were input into EndNote 7X (Thomson Reuters, New York, NY, USA) software to manage the data.

### 2.4 Assessment of risk of bias

Two investigators [Wen Jia and Chuan Wang] independently evaluated the risk of bias in the included RCTs according to the Cochrane Collaborations tool.^[16]^ The following seven factors were evaluated: (1) random sequence generation; (2) allocation concealment; (3) blinding of the participants and personnel; (4) blinding of outcome assessment; (5) incomplete outcome data; (6) selective reporting; and (7) other bias. Each factor was categorized as low risk, high risk or unclear. Any disagreements were resolved by a third author [Ying Yin].

### 2.5 Statistical analysis

We conducted all statistical analyses with RevMan V.5.3.3 software. The indicators of semen quality were expressed as the standard mean difference (SMD) with 95% confidence intervals (CIs). We used a random-effects model or a fixed-effects model for the metaanalysis of the included studies based on the existence of heterogeneity between the study results. Heterogeneity was assessed using Cochrane's Q test and I2 statistics.^[17]^ We used a funnel plot to assess publication bias when 10 or more trials were included in a meta-analysis, and approximately symmetrical funnel plots were considered indicative of a low risk of bias.^[18]^

## 3 Results

### 3.1 Study screening and characteristics of the included studies

**Figure 1:**
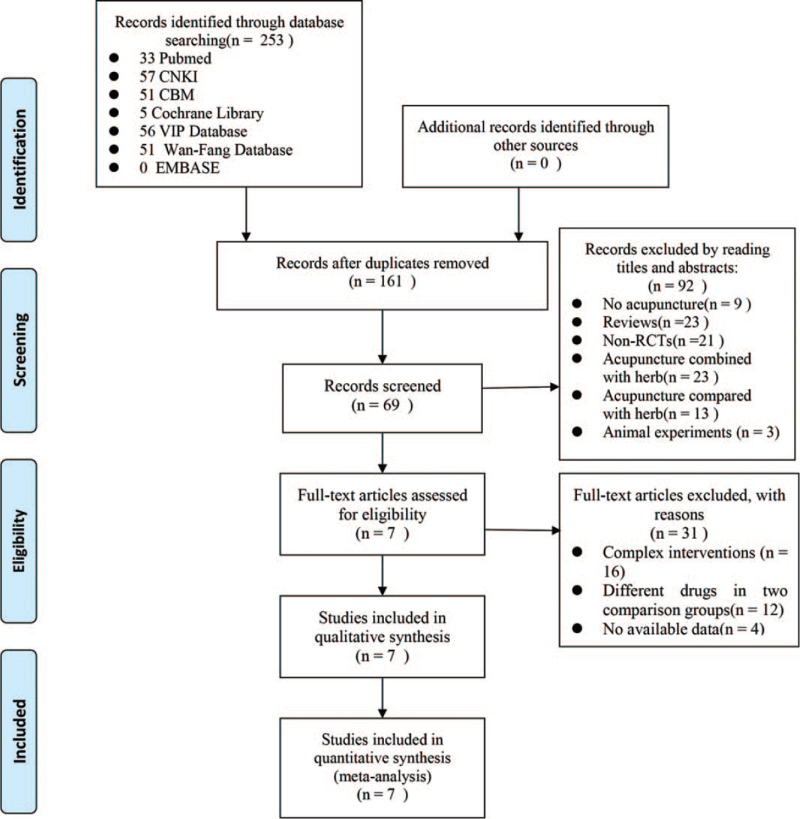
Flow chart of the trial selection process. RCT = randomized controlled trial.

A total of 253 records were identified from 7 electronic databases; after 122 duplicates were excluded, 131 records were considered for screening. A total of 92 irrelevant records were excluded after screening the titles and abstracts. Thirty-two records were excluded after the full-text screening. Finally, 7 RCTs involving 527 patients were eligible for inclusion.^[19–25]^Figure 1 shows the flow chart of the study searches. The characteristics of the included studies are shown in Table 2.

**Table 2:** Characteristics of the included studies on acupuncture for oligospermia and asthenozoospermia.

Study (yr)	Sample (n): age	Duration of disease	Experimental group	Control group	Treatment (T)Follow-up (F)	Outcome measurements	AdverseEvents (n)
Gurfinkel et al (2003)^[19]^	E (8): mean 33.4 yrC (10): mean 31.6 yr	E: median 7.6 yrC: median 6.1 yr	Manual acupuncture and moxa	Placebo acupuncture	T: 10 wkF: NR	Percentage of normal-form sperm	NR
Dieterle et al (2010)^[20]^	E (24): NRC (28):NR	NR	Manual acupuncture	Placebo acupuncture	T: 6 wkF: 2 mo	1.Sperm motility2.Sperm concentration3.Semen volume	No adverse events
Zhang et al (2016)^[21]^	E (30): 31.68 ± 1.85 yrC (30):32.15 ± 1.45 yr	E: 2.45 ± 1.25 yrC: 2.35 ± 1.46 yr	Fire needle and manual acupuncture	Clomiphene	T: 12 wkF:NR	Sperm motility	NR
Sun et al (2016)^[22]^	E (42): 32 ± 3 yrC (40): 31 ± 3 yr	E: 6.4 ± 0.5 yrC: 6.3 ± 0.3 yr	Manual acupuncture	Placebo acupuncture	T: 12 wkF:NR	1.Sperm motility2.Sperm concentration	NR
Wang et al (2016)^[23]^	E (37): 26.38 ± 3.54 yrC (38): 26.16 ± 3.16 yr	E: 35.62 ± 5.61 moC: 34.84 ± 4.65 mo	Manual acupuncture	Vitamin E and vitamin C	T: 3 moF: NR	1.Sperm motility2.Sperm concentration	E: 1 case with fainting during acupunctureC: 0
Li et al (2017)^[24]^	E1 (40): 30.43 ± 3.27 yrE2 (40): 32.14 ± 3.37 yrC (40): 29.95 ± 3.32 yr	E1: 4.66 ± 1.97 yrE2: 4.75 ± 2.35 yrC: 5.05 ± 2.63 yr	E1 Tamoxifen+TEASE2 TEAS	Tamoxifen	T: 8 wkF: NR	1. Sperm motility2.Sperm concentration	NR
Liu et al (2017)^[25]^	E1 (40): NRE2 (40): NRC (40): NR	NR	E1 L-carnionc+moxaE2 Moxa	L-carnionc	T: 3 morF:NR	1.Sperm motility2.Sperm concentration3.Semen volume	NR

### 3.2 Study description

All studies were published from 2003 to 2017; 5 trials were performed in China,^[21–25]^ 1 was performed in Brazil^[19]^ and 1 was performed in Germany.^[20]^ Five studies were two-arm trials,^[19–23]^ and 2 were three-arm trials.^[24,25]^

#### 3.2.1 Patients

Seven studies involving 527 patients were included, and all of the participants were included in the statistical analysis.

#### 3.2.2 Acupuncture intervention

All treatment protocols were based on traditional Chinese medicine theory and the clinical experience of the acupuncturists. Three trials used manual acupuncture alone,^[20,22,23]^ 1 trial used the combination of manual acupuncture and moxa,^[19]^ 1 trial used the combination of manual acupuncture and fire needle,^[21]^ 1 trial used the combination of transcutaneous electrical acupoint stimulation and tamoxifen,^[24]^ and another trial used the combination of moxa and L-carnitine.^[25]^ The acupoints for each trial are shown in Table 3.

**Table 3:** Selected acupoints of each study.

Study (year)	Acupoints
Gurfinkel et al(2003)^[19]^	Qichong (ST 30), Taixi (KI 3), Zusanli (ST 36), Hegu (LI 4), Sanyinjiao (SP 6), Gongsun (SP 4), Taichong (LR 3), Neiguan (PE 6)
Dieterle et al(2010)^[20]^	Zusanli (ST 36), Sanyinjiao (SP 6), Taixi (KI 3), Taichong (LR 3), Shenshu (BL 23), Ciliao (BL 32), Guilai (ST 29), Xuehai (SP 10), Guanyuan (RN 4), Baihui (DU 20)
Zhang et al (2016)^[21]^	Shenshu (BL 23), Zusanli (ST 36), Taixi (KI 3), Taichong (LR 3), Baihui (DU 20)
Sun et al (2016)^[22]^	Guanyan (RN 4), Qihai (RN 6), Baihui (DU 20), Zusanli (ST 36), Yinlinquan (SP 9), Sanyinjiao (SP 6)
Wang et al (2016)^[23]^	Guanyan (RN 4), Qihai (RN 6), Sanyinjiao (SP 6), Zusanli (ST 36), Fenglong (ST 40), Shenshu (BL 23), Mingmen (DU 4), Taixi (KI 3), Ciliao (BL 32)
Li et al (2017)^[24]^	Guanyan (RN 4), Sanyinjiao (SP 6), Zusanli (ST 36), Shenshu (BL 23), Yongquan(KI 1)
Liu et al (2017)^[25]^	Dazhui (DU 14), Taodao (DU 13), Shenzhu (DU 12), Shendao (DU 11), Lingtai (DU 10), Zhiyang (DU 9), Jinsuo (DU 8), Zhongshu (DU 7), Jizhong(DU 6), Xuanshu (DU 5), Mingmen (DU 4), Yaoyangguan (DU 3)

#### 3.2.3 Control interventions

Three studies compared acupuncture to placebo acupuncture,^[19,20,22]^ and 4 studies compared acupuncture to conventional medications (including clomiphene, vitamin E, vitamin C, tamoxifen, and L-carnitine).^[21,23–25]^

#### 3.2.4 Outcome measures

All studies evaluated semen samples according to the World Health Organization standard_._^[5]^ Six studies measured sperm motility,^[20–25]^ 5 studies measured sperm concentration,^[20,22–25]^ 2 studies measured semen volume,^[19,24]^ and 1 study measured the percentage of normal-form sperm.^[19]^

#### 3.2.5 Risk of bias within studies

**Figure 2:**
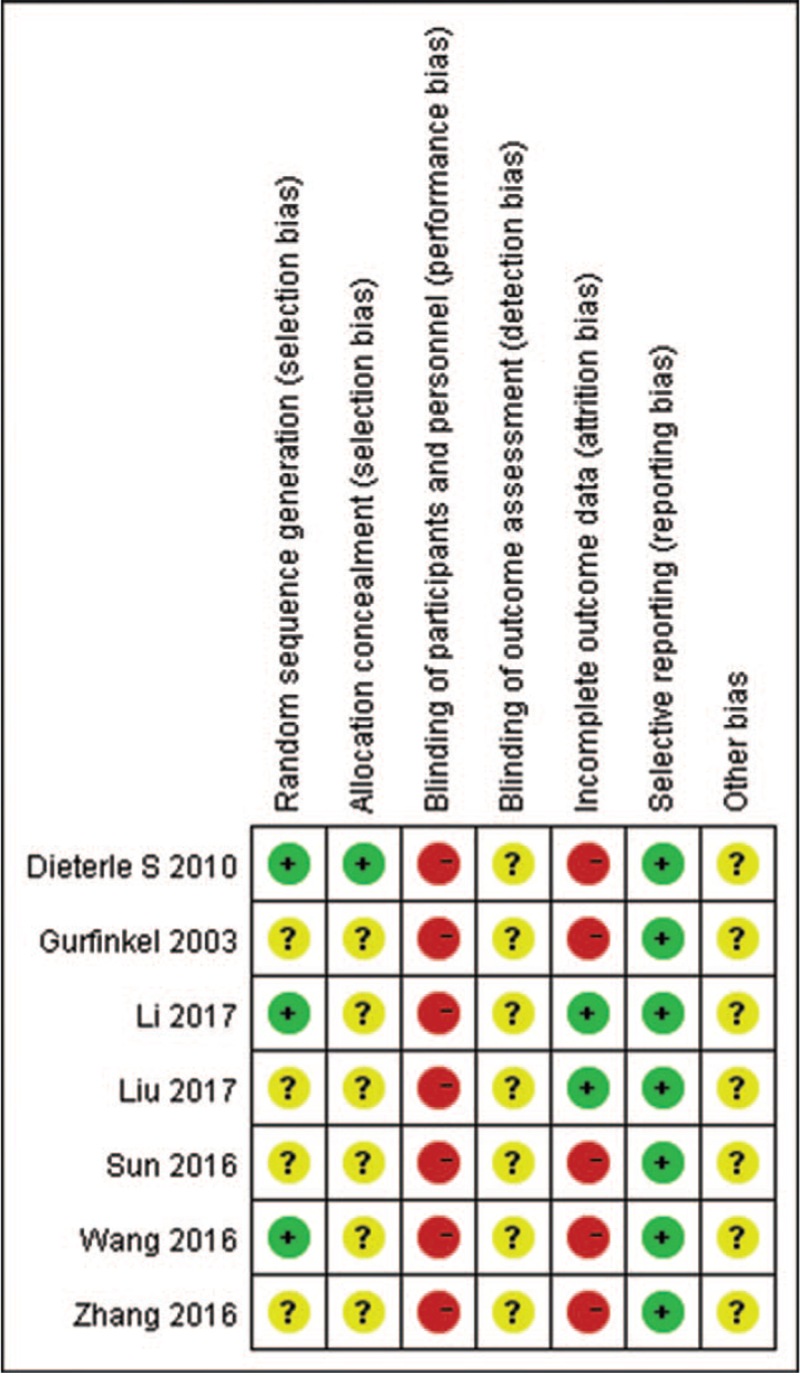
Summary of the risk of bias of the included trials.

All of the studies mentioned randomization, but only 3 studies reported adequate sequence generation,^[20,23,24]^ 2 studies used the random number table,^[23,24]^ and 1 used a computer-based random number generator.^[20]^ One study reported details about allocation concealment, which ensured that all patients and study personnel were blinded to group assignment for the duration of the study.^[20]^ Because of the nature of acupuncture, acupuncturists cannot be blinded, but 3 studies used placebo acupuncture as a control intervention that ensured blinding of the patients.^[19,20,22]^ No studies reported blinding of outcome assessors. Five studies reported dropouts without providing any reasons.^[19–23]^ Details on the risk of bias are shown in Figure 2.

### 3.3 Effects of acupuncture

#### 3.3.1 Acupuncture versus placebo acupuncture

##### 3.3.1.1 Sperm motility (%)

**Figure 3:**
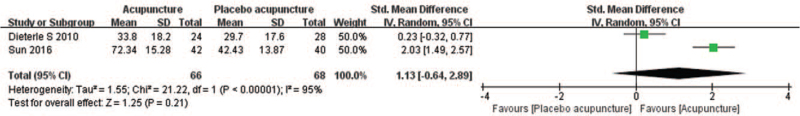
Meta-analysis of the sperm motility of the acupuncture versus placebo acupuncture. CI = confidence interval, SD = standard deviation.

Two studies involving 134 participants reported sperm motility.^[20,22]^ The meta-analysis of the 2 studies showed no significant differences between the acupuncture group and the placebo acupuncture group (standard mean difference [SMD] = 1.13, 95% confidence interval [CI]: −0.64 to 2.89), but the heterogeneity (*I*^2^ = 95%, *P* < .0001) was high (Fig. 3).

##### 3.3.1.2 Sperm concentration (million/mL)

**Figure 4:**
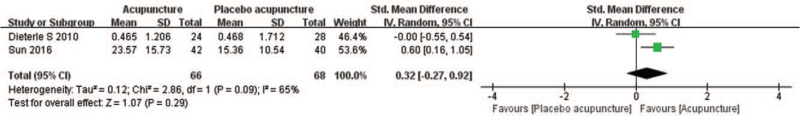
Meta-analysis of the sperm concentration of the acupuncture versus placebo acupuncture. CI = confidence interval, SD = standard deviation.

Two studies involving 134 participants reported sperm concentration.^[20,22]^ A meta-analysis of the 2 studies showed no significant differences between the acupuncture group and the placebo acupuncture group (SMD = 0.32, 95% CI: −0.27 to 0.92), but the heterogeneity (*I*^2^ = 65%, *P* = .09) was high (Fig. 4).

##### 3.3.1.3 Semen volume (mL)

Only 1 study involving 52 participants found no difference in semen volume between acupuncture and placebo acupuncture (3.7 ± 1.4 versus 3.8 ± 1.6).^[20]^

##### 3.3.1.4 Percentage of normal-form sperm (%)

Only 1 study involving 18 participants found that manual acupuncture and moxa were more effective than placebo acupuncture in improving the percentage of normal-form sperm (60.00 ± 22.04 versus 35.38 ± 28.78).^[19]^

#### 3.3.2 Acupuncture versus drugs

##### 3.3.2.1 Sperm motility (%)

**Figure 5:**
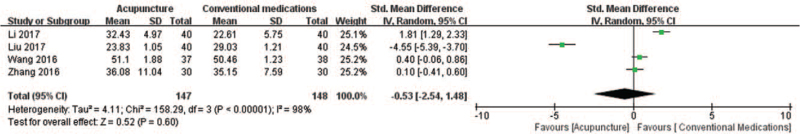
Meta-analysis of the sperm motility of the acupuncture versus drugs. CI = confidence interval, SD = standard deviation.

Four studies involving 295 participants reported no significant difference between acupuncture and conventional medications in improving sperm motility (SMD = −0.53, 95% CI: −2.54 to 1.48); however, the heterogeneity (*I*^2^ = 98%, *P* < .0001) was high (Fig. 5).^[21,23–25]^

##### 3.3.2.2 Sperm concentration (million/mL)

**Figure 6:**
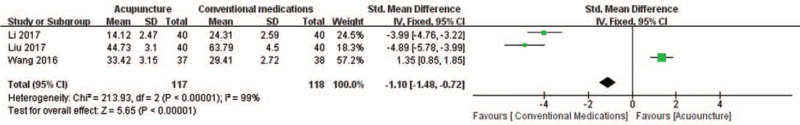
Meta-analysis of the sperm concentration of the acupuncture versus drugs. CI = confidence interval, SD = standard deviation.

Three studies involving 235 participants reported that the conventional medicine group had a significant difference in sperm concentration compared to the acupuncture group (SMD = −1.10, 95% CI: −1.48 to −0.72), but the heterogeneity (*I*^2^ = 99%, *P* < .0001) was high (Fig. 6).^[23–25]^

##### 3.3.2.3 Semen volume (mL)

Only the 1 study involving 80 participants found that moxa was more effective than L-carnitine in increasing the semen volume (4.02 ± 0.24 versus 3.26 ± 0.36).^[25]^

#### 3.3.3 Acupuncture plus conventional medication versus the same conventional medication

##### 3.3.3.1 Sperm motility (%)

**Figure 7:**
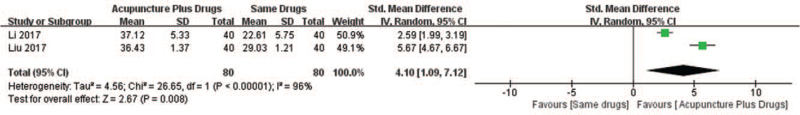
Meta-analysis of the sperm motility of the acupuncture plus drugs versus the same drugs. CI = confidence interval, SD = standard deviation.

Two studies involving 160 participants found that acupuncture plus conventional medication was significantly more effective than conventional medication alone in improving sperm motility (SMD = 4.10, 95% CI: 1.09–7.12), but the heterogeneity (*I*^2^ = 96%, *P* < .0001) was high (Fig. 7).^[24,25]^

##### 3.3.3.2 Sperm concentration (million/mL)

**Figure 8:**
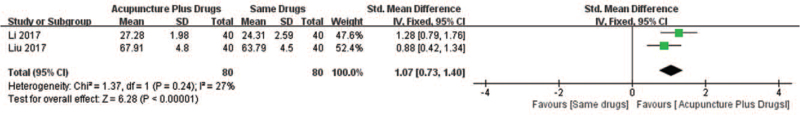
Meta-analysis of the sperm concentration of the acupuncture plus drugs versus the same drugs. CI = confidence interval, SD = standard deviation.

Two studies involving 160 participants found that acupuncture plus conventional medication was significantly more effective than conventional medication alone in improving the sperm concentration (SMD = 1.07, 95% CI: 0.739–1.40), and the heterogeneity (*I*^2^ = 27%, *P* = .24) was low (Fig. 8).^[24,25]^

##### 3.3.3.3 Semen volume (mL)

Only 1 study involving 80 participants found no difference in semen volume between moxa plus L-carnitine and L-carnitine alone (3.23 ± 0.28 versus 3.26 ± 0.36).^[25]^

### 3.4 Adverse events

In 1 trial, 1 case of fainting during acupuncture was reported.^[23]^ In another trial, all patients had no adverse events during treatment.^[19]^ Five trials did not report whether adverse events occurred during treatment.^[19,21,22,24,25]^

## 4 Discussion

This systematic review included 7 RCTs involving 527 patients. Five kinds of acupuncture were identified in the treatment of oligospermia and asthenozoospermia, including manual acupuncture, moxa, manual acupuncture plus fire needle, manual acupuncture plus moxa, and transcutaneous electrical acupoint stimulation. The results of the meta-analysis showed no difference in semen quality improvements between acupuncture alone and placebo acupuncture or conventional medications. Acupuncture plus conventional medications may be more effective than conventional medication alone in improving semen quality, but the methodological quality of the included studies was very low, and the heterogeneity was high; thus, the current evidence was insufficient to draw definitive conclusions.

Some observational studies have suggested that acupuncture might have an effect on male infertility,^[26]^ and some nonrandomized control studies found that the sperm concentrations and the percentage of ultramorphologically normal sperm were significantly higher after acupuncture compared with the waiting list group.^[14,27]^ Acar et al^[28]^ suggested that acupuncture might improve testicular perfusion by stimulating the nerve. Siterman et al^[29]^ suggested that acupuncture might reduce genital inflammatory reactions by enhancing immune responses. However, the mechanism of acupuncture related to sperm quality is not clear.

In this systematic review, only 7 studies were included in the statistical analysis. Three studies reported no significant differences between acupuncture and placebo acupuncture in improving sperm motility (SMD = 1.13, 95% CI: −0.64 to 2.89),^[20,22]^ the sperm concentration (SMD = 0.32, 95% CI: −0.27 to 0.92),^[20,22]^ or semen volume (3.7 ± 1.4 versus 3.8 ± 1.6).^[20]^ Four studies found no significant difference between acupuncture and conventional medication in improving sperm motility (SMD = −0.53, 95% CI: −2.54 to 1.48),^[21,23–25]^ while 3 studies reported that conventional medication might be more effective than acupuncture in improving the sperm concentration (SMD = −1.10, 95% CI: −1.48 to −0.72).^[23–25]^ Therefore, the current evidence does not support acupuncture as an effective treatment for oligospermia and asthenozoospermia, which differs from the result of a previous systematic review.^[15]^ The main reason for this phenomenon is that the previous systematic review included RCTs that applied acupuncture plus an herbal mixture for oligospermia and asthenozoospermia and used the same herbal mixture as the control groups; however, the herbal mixture was not recommended for the treatment of oligospermia and asthenozoospermia by the clinical practice guidelines on infertility. Therefore, the research design of the previous review was not appropriate, and the results of that review were not objective.

Two studies found that acupuncture plus conventional medication was significantly more effective than conventional medication alone in improving sperm motility (SMD = 4.10, 95% CI: 1.09–7.12),^[24,25]^ which seems to imply that acupuncture can help to enhance the effects of conventional drugs, but the heterogeneity among these studies was too high to make robust conclusions. The only statistically significant improvement with a low level of heterogeneity (*P* = .24) seems to be the semen concentration (SMD = 1.07, 95% CI: 0.739–1.40),^[24,25]^ and the result is same as the result of the previous study,^[27]^ reflecting that acupuncture may have potential therapeutic value in sperm disorders. However, because of high statistical heterogeneity in other results, this does not support acupuncture as an effective treatment for oligospermia and asthenozoospermia.

One trial reported 1 case of fainting during acupuncture,^[23]^ and another trial reported that all patients had no adverse events during treatment.^[20]^ However, most trials did not report whether adverse events occurred during treatment; thus, whether acupuncture is a safe treatment for oligospermia and asthenozoospermia is uncertain.

The quality of the included trials was low, and the lack of blinding and randomization increased the selection bias and performance bias. Incomplete information and an unreasonable loss of patients led to a confounding bias in these results. Small sample sizes complicated determination of firm conclusions regarding the study outcomes, and the results of this study may change as more trials are conducted.

## 5 Conclusion

The results of this study suggest that acupuncture alone has no clear superiority in improving sperm quality compared with conventional medications or placebo acupuncture, so acupuncture is not recommended for improving the sperm quality and count of healthy individuals. Adjuvant acupuncture may enhance the effect of conventional medications, but the study heterogeneity was too high to make robust conclusions. The current evidence does not support acupuncture as an effective treatment for oligospermia and asthenozoospermia, more large-scale, high-quality clinical trials will be needed in the future.

### 5.1 Uncited references

^[16–18]^.

## Author contributions

Wen Jia and Ying Yin conceived the study. Wen Jia and Chuan Wang reviewed studies for inclusion, assessed the included studies, extracted data, completed the first draft and edited the review. Ying Yin arbitrated in cases of disagreement and ensured the absence of errors. All authors approved the final manuscript.

**Data curation:** Chuan Wang.

**Writing – original draft:** Wen Jia, Chuan Wang.

**Writing – review & editing:** Wen Jia, Ying Yin.
